# Enhancement of complement-dependent cytotoxicity by linking factor-H derived short consensus repeats 19-20 to CD20 antibodies

**DOI:** 10.3389/fimmu.2024.1379023

**Published:** 2024-07-22

**Authors:** Lena Prantl, Philipp Heider, Lisa Bergmeister, Katharina Calana, Jan-Paul Bohn, Dominik Wolf, Zoltan Banki, Andreas Bosch, Maximilian Plach, Georg Huber, Silke Schrödel, Christian Thirion, Heribert Stoiber

**Affiliations:** ^1^ Institute of Virology, Innsbruck Medical University, Innsbruck, Austria; ^2^ Department of Internal Medicine V, Hematology & Oncology, Comprehensive Cancer Center Innsbruck (CCCI), Tyrolean Cancer Research Institute (TKFI), Medical University of Innsbruck, Innsbruck, Austria; ^3^ 2bind GmbH, Regensburg, Germany; ^4^ Sirion Biotech GmbH, Gräfelfing, Germany; ^5^ Lysomab GmbH, Schwaz, Austria

**Keywords:** CLL, complement-dependent cytotoxicity, ofatumumab, rituximab, obinutuzumab, complement factor H, SCR

## Abstract

Antibody-mediated complement-dependent cytotoxicity (CDC) on malignant cells is regulated by several complement control proteins, including the inhibitory complement factor H (fH). fH consists of 20 short consensus repeat elements (SCRs) with specific functional domains. Previous research revealed that the fH-derived SCRs 19–20 (SCR1920) can displace full-length fH on the surface of chronic lymphocytic leukemia (CLL) cells, which sensitizes CLL cells for e.g. CD20-targeting therapeutic monoclonal antibody (mAb) induced CDC. Therefore, we constructed lentiviral vectors for the generation of cell lines that stably produce mAb-SCR-fusion variants starting from the clinically approved parental mAbs rituximab, obinutuzumab and ofatumumab, respectively. Flow-cytometry revealed that the modification of the mAbs by the SCRs does not impair the binding to CD20. Increased *in vitro* lysis potency compared to their parental mAbs was corroborated by showing specific and dose dependent target cell elimination by CDC when compared to their parental mAbs. Lysis of CLL cells was not affected by the depletion of NK cells, suggesting that antibody-dependent cellular cytotoxicity plays a minor role in this context. Overall, this study emphasizes the crucial role of CDC in the elimination of CLL cells by mAbs and introduces a novel approach for enhancing CDC by directly fusing fH SCR1920 with mAbs.

## 1 Introduction

Anti-CD20 monoclonal antibodies (mAbs) have become key-components in the treatment of chronic lymphocytic leukemia (CLL) facilitating deep and long-lasting remissions in most patients (1). However, although many reports have demonstrated the complementary efficacy of these mAbs when combined with chemotherapy (i.e. R-Benda or FCR) or targeted compounds (i.e. BTKis or venetoclax), uncertainty remains concerning the mode of action responsible for efficient elimination of CLL cells. Increasing evidence based on primary tumor cells, correlative analysis in clinical trials and murine models suggests that the cytotoxic effects of the anti-CD20 mAbs mainly rely on immune effector functions, such as antibody-dependent cellular cytotoxicity (ADCC), phagocytosis, or complement-dependent effector functions (2, 3). According to their binding and effector properties, CD20 mAbs are divided into type I and type II antibodies (4). Rituximab (RTX) and ofatumumab (OFA), which are classified as type I, are able to redistribute CD20 into lipid rafts (4). The clustering of antibody Fc portions leads to increased levels of complement dependent cytotoxicity (CDC) *via* the classical activation pathway (5). This CD20 redistribution and enhanced CDC activation is not observed in type II antibodies, like obinutuzumab (OBI), which is the most frequently used mAb in CLL therapy. Type II mAbs favor the induction of ADCC and direct induction of non-apoptotic cell death (5).

In contrast to OBI, both RTX and OFA can activate the complement system quite efficiently upon binding to malignant B cells (2, 6). However, CDC induction on B cell lymphoma cells is impaired by several complement control proteins including complement factor H (fH) (7). fH consists of 20 short consensus repeat (SCR) elements, which harbor specific functional domains. SCRs 19–20 (SCR1920) facilitate the binding to cell surfaces by recognizing anionic structures as well as C3b and C3d, while SCRs 1–4 are able to bind complement C3b and inhibit the deposition of C3b, and impairs the activity of C3-convertase and C5-convertase (7, 8). Previous research has revealed that the fH-derived SCRs 19–20 can displace full-length fH from cell surfaces, eliminating the complement inhibitory function of full-length fH (9, 10). This reduction of surface-bound fH sensitizes cells for CDC induced by mAbs targeting CD20. Thus, we produced mAb-SCR conjugates of RTX, OFA and OBI and the parental mAbs by lentiviral transduction in cell culture, purified them by affinity chromatography and finally tested them by *in vitro* lysis experiments. Our results indicate that the anti-CD20-SCR1920 conjugates significantly improved the efficacy of the mAbs by enhanced induction of CDC, which appears to be NK cell-independent in our experimental setting suggesting preferential killing by complement-mediated target cell lysis. In summary, we provide clear evidence that modifying mAbs to enhance CDC improves the efficacy of targeted cell killing, which may set the stage for future *in vivo* validation in preclinical modes whether this also translates into deeper remission rates (i.e. increased significant minimal residual disease negativity rates, which are linked to improved outcome in various diseases) (11).

## 2 Materials and methods

### 2.1 Isolation and culture of PBMCs from patients with CLL

This study was approved by the Ethics Committee of the Medical University of Innsbruck (approval no. 1154/2019). After receiving informed consent, peripheral blood mononuclear cells (PBMCs) were obtained from heparinized blood of anti-CD20 antibody therapy naïve CLL patients at the University Clinics Innsbruck, Department of Internal Medicine V, Hematology & Oncology. PBMCs were isolated by density gradient centrifugation with Pancoll human (PAN-Biotech, Aidenbach, Germany) as recommended by the manufacturer. PBMCs were cultured in RPMI-1640 medium (Sigma-Aldrich, St. Louis, MO, USA), supplemented with 10% heat-inactivated fetal bovine serum (FBS; PAN-Biotech), 1 mM L-glutamine (Thermo Fisher Scientific, Waltham, MA, USA) and stimulated over night with 0.5 µg/mL bacterial lipopolysaccharide (LPS from *E. coli* 026:B6; Sigma-Aldrich).

### 2.2 Preparation of RTX-, OBI- and OFA-SCR-Ab constructs in HEK293 cells

HEK293 (CRL-1573) cells (ATCC, Manassas, VA, USA) were cultured in high glucose DMEM (Sigma-Aldrich) supplemented with 10% FBS, 2 mM L-glutamine. Lentiviral expression vectors were constructed to encode antibody (Ab) light or heavy chains (LC or HC of RTX, OBI and OFA, respectively) and sequences for the complement fH (UniProt accession number P08603) derived SCRs 19–20, 16–17 or 11–12 (SCR1920, SCR1617 and SCR1112, respectively) were used for transfection in a two vector approach. Stably transduced expression cell lines were generated by antibiotic selection. Monoclonal Ab-SCR conjugates were purified by affinity chromatography using a prepacked HiTrap MabSelect PrismA protein A column (Cytiva, Marlborough, MA, USA) connected to a peristaltic pump, according to manufacturer’s instructions. Elution was initiated by a shift toward acidic pH (0.1 M glycin-HCl, pH 3.0 elution buffer) and fractionated eluates were immediately neutralized to a pH of 6.5 by adding pre-determined amounts of 1 M Tris-HCl (pH 9.0) neutralization buffer. Antibody concentrations of eluted fractions were measured photometrically.

### 2.3 Sodium Dodecyl Sulfate Polyacrylamide Gel Electrophoresis (SDS-PAGE) and Western Blot

Appearance and purity of fractions were analyzed under reducing conditions with SDS-PAGE and subsequent Western blot. Two hundred ng of antibody were loaded on 10% SDS-polyacrylamide gels. After electrophoresis, proteins were transferred to nitrocellulose membranes (Bio-Rad Laboratories, Hercules, CA, USA) by semidry Western blot. Detection was performed with a polyclonal goat Anti-Human IgG (H+L) POX antibody (1:5,000; Jackson ImmunoResearch, West Grove, PA, USA) and ECL substrate at an ImageQuant LAS 4000 (GE Healthcare, Chicago, IL, USA) device.

### 2.4 Complement dependent cytotoxicity (CDC) assay of CLL PBMCs

CDC assays were performed as described previously (9, 10). In brief, 2x10^5^ CLL PBMCs were mixed with different concentrations of purified mAb-SCR ranging from 10–100 µg/mL. Normal human serum (NHS), obtained and pooled from 10 healthy donors, was at a final concentration of 20% in a total assay volume of 125 µL per sample. A heat inactivated fraction of this pooled normal human serum (hiNHS; incubation at 56°C for 1 h to inactivate complement) was used as control. In some experiments recombinant fH-derived SCR1920 peptide (1200 µg/mL) was added in combination with OFA (20 µg/mL) to compare the effect of SCR peptides with the directly linked constructs. For all lysis experiments, samples were incubated for 1 h at 37°C and 5% CO_2_. Following incubation, propidium iodide (PI; Sigma-Aldrich) was added, enabling the discrimination of dead and viable cells by flow-cytometry. Samples were measured for 60 sec at constant flow rate on a FACS Canto II flow-cytometer (BD Biosciences, Franklin Lakes, NJ, USA). The percentages of surviving cells were calculated relative to the hiNHS control sample which was defined as 100% survival. The survival rates were calculated according to the following formula: percent of survival = 100% * count of viable cells in treated sample/count of viable cells in hiNHS control sample.

### 2.5 Flow-cytometry

PBMCs from CLL patients were incubated with RTX or RTX-SCR constructs under standard CDC assay conditions as described above (20% NHS or hiNHS, 1 h incubation at 37°C, 5% CO_2_). Prior to analysis by flow-cytometry, cells were stained with allophycocyanin (APC)-conjugated anti-human CD19 (clone HIB19; BioLegend, San Diego, CA, USA) and PI. In total, 30 000 events per sample were analyzed on a FACS Canto II flow-cytometer and analyzed with FlowJo™ vX software (BD Life Sciences, Ashland, OR, USA). PI-negative, viable cells were gated into CD19-positive (B cells) and CD19-negative (non-B cells) cells. The survival rates for B cells and non-B cells were calculated relative to the hiNHS control sample.

The antigen binding capacity of RTX constructs or the CD20 expression of CLL patients were determined by incubating 2x10^5^ PBMCs with 5 µg antibody or antibody-construct for 30 min at 4°C. After washing, cells were stained with polyclonal rabbit anti-human fluorescein isothiocyanate (FITC) conjugated IgG, specific for gamma-chains (Agilent Technologies, Santa Clara, CA, USA) at 4°C for 30 min and analyzed as described above.

The complement deposition on B cells and non-B cells was determined by staining with APC-conjugated anti-human CD19 and polyclonal rabbit anti-human C3c complement/FITC (Agilent Technologies, Santa Clara, CA, USA).

### 2.6 NK cell depletion or isolation from PBMCs

PBMCs were depleted from NK cells by REAlease^®^ CD56 MicroBead Kit (Miltenyi Biotec, Bergisch Gladbach, Germany) according to manufacturer’s recommendations. Briefly, PBMCs were mixed and incubated with REAlease^®^ CD56 Biotin antibodies. REAlease^®^ anti-Biotin Micro-Beads were added, mixed and incubated with antibody labeled cells. A Miltenyi LS-Column was placed into a magnetic separator and rinsed with separation buffer. The cell suspension was loaded and the flow-through containing the unlabeled PBMCs was collected. The NK cell depleted flow-through was washed two times in RPMI-1640 medium and was subsequently used in CDC assays as described above. The column was washed 3 times with separation buffer. The column was removed from the magnetic separator and bound NK cells were eluted. The REAlease complex was removed by the addition of REAlease Release Reagent. Effectivity of NK cell depletion or isolation was confirmed by flow-cytometry using BD multitest CD3 FITC/CD16 PE + CD56 PE/CD45 PerCP/CD19 APC (BD Biosciences) according to manufacturer’s recommendations.

### 2.7 Statistical analysis

Statistical analyses were performed using Prism version 9.2.0 (GraphPad Software, Boston, MA, USA). The effects of mAb-SCR on CLL PBMCs are presented as mean with standard deviation. Statistical significance was calculated by Student’s t-test or one-way analysis of variance (ANOVA) with subsequent Tukey’s multiple comparisons test. P-values < 0.05 were considered to indicate statistical significance.

## 3 Results

### 3.1 mAb-SCR constructs derived from HEK293 cells exhibit uniform appearance and demonstrate stability

**Figure 1:**
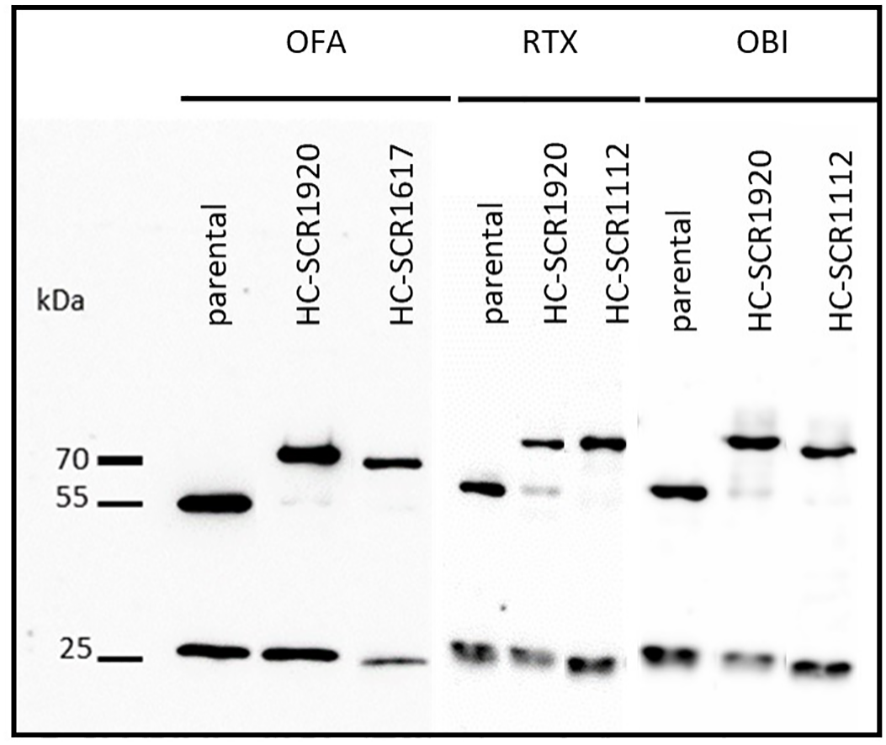
Western blot analysis of CD20-mAbs OFA, RTX and OBI conjugated to human SCR1920 or SCR1112/SCR1617 via the antibody heavy chain. Antibody constructs were purified by Protein A affinity chromatography (HiTrap MabSelect PrismA, Cytiva) from HEK-293 cell culture supernatant and eluted acidically. Concentration was adjusted to 20 µg/mL for separation by 10%, reducing SDS-PAGE and analysis by Western blot. For the detection of antibody constructs, POX-conjugated goat anti-human IgG (HC+LC) antibody (1:5,000; Jackson ImmunoResearch) and ECL substrate were used. Specific bands were expected at 25, 50 and 66 kDa for LC, HC and SCR-conjugated-HC, respectively.

Cells were transduced with a combination of two lentiviral vectors, encoding antibody light chain (LC) sequences or the corresponding antibody heavy chain (HC) sequences with or without SCR-conjugations (SCR11–12, SCR16–17 or SCR19–20). SCR-conjugations were located at the HC of the mAbs. The presence of parental mAbs or antibody-SCR conjugates was confirmed by SDS-PAGE under reducing conditions and a subsequent Western Blot analysis. All constructs were diluted to a concentration of 20 µg/mL aiming the visualization of comparable band intensities of the unmodified LC. The detection of HC and LC was achieved by POX-conjugated goat anti-human IgG (H+L) antibody and ECL substrate. Bands for LC, HC and SCR-conjugated HC were expected and appeared at 25 kDa, 55 kDa and 70 kDa, respectively (**Figure 1**). Comparison of parental mAbs with the SCR-fusion constructs by dynamic light scattering (DLS) and nano differential scanning fluorimetry (nanoDSF) revealed that neither the colloidal nor thermal stability of the produced mAbs were affected by the addition of the SCR (**Supplementary Figures 1**, **2**).

### 3.2 In-house produced antibodies induce lysis in a comparable efficacy as commercial antibodies

**Figure 2:**
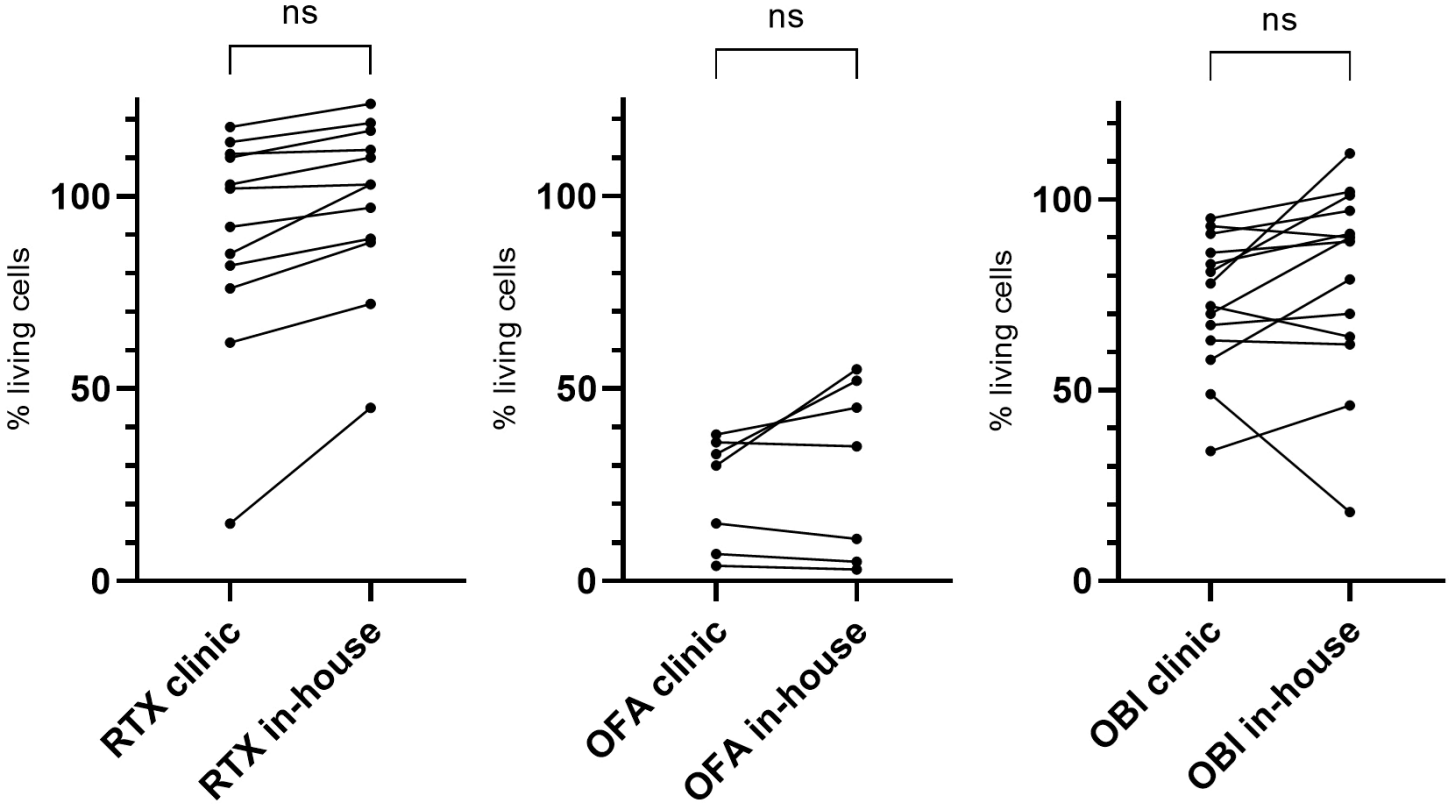
Lysis induced by clinically used CD20 mAbs and in-house produced CD20 mAbs does not differ significantly in standard CLL lysis tests. PBMCs from different CLL patients were treated with CD20 mAbs (RTX, OFA or OBI) at 50 µg/mL. Twenty percent NHS was added as a source for complement. PI-negative, viable cells were counted by flow-cytometry for 60 sec at a constant flow rate. Survival rates were calculated relative to a hiNHS sample. Corresponding measurements from one patient are connected by a line. Ratio paired t-tests were calculated to compare the means of corresponding samples. ns, not significant; n(RTX)=12, n(OFA)=7, n(OBI)=13.

Clinically used CD20 mAbs and their in-house produced and purified equivalents were tested side by side in standard CLL PBMC lysis tests on patients who did not respond to treatment with parental mAbs *in vitro* (n(RTX)=7; n(OFA)=12; n(OBI)=13. 2x10^5^ cells were treated with 50 µg/mL antibody and 20% NHS for 1 h at 37°C, 5% CO_2_. Cell viability was determined by PI staining and flow-cytometry measurement for 60 sec per sample at a constant flow rate. Percent living cells were calculated relative to antibody-untreated and hiNHS containing control samples. The response to the antibody treatment varied between patients. However, within a single patient, the differences between the clinically used mAbs and the in-house antibodies were minor and lacked overall significance (**Figure 2**). For this reason, in the subsequent experiments only in-house mAbs were used, given their uniform production and formulation processes of the parental mAbs and the mAb-SCR constructs allowing comparability between the mAbs used in the study.

Additionally, it was tested if the antigen binding capacity of RTX-SCR constructs is impaired. PBMCs of 3 different CLL patients were treated with 5 µg of the respective antibody in the absence of NHS. The surface-bound RTX-constructs were detected with an anti-human IgG-FITC conjugated secondary antibody and the mean fluorescence intensities (MFI) of the FITC-positive populations were determined by flow-cytometry. As patients differ in their CD20 surface expression, the MFIs varied between the three tested PBMC samples. The means and standard deviations were calculated and the statistical significance was analyzed by ordinary one-way ANOVA and a subsequent Tukey’s multiple comparisons test (**Supplementary Figure 3**). The antigen binding capacity did not differ significantly between the clinically used RTX, the in-house produced RTX, RTX-SCR1920 and RTX-SCR1112. Hence, the in-house production process and SCR-conjugations did not change the CD20 binding capacity significantly and thus excludes a potential cause for variations in target cell lysis.

### 3.3 Fusion of SCR1920 with CD20 mAbs increases their cytotoxicity against CLL target cells significantly

It was recently shown that the addition of SCR1920 peptides to CD20 targeting mAbs like RTX or OFA significantly enhanced the lysis capacity of the tested mAbs (9, 10). However, the *in vivo* treatment with an excess of SCR1920 would presumably be problematic because of unspecific, competitive displacement of full-length factor H from host cell surfaces and subsequent undirected, generalized complement attacks. To achieve a target specific factor H displacement and complement activation, SCRs were coupled to CD20-directed mAbs. SCR1112- or SCR1617-constructs were produced and tested as negative controls, not comprising any relevant functional domains of fH (7).

**Figure 3:**
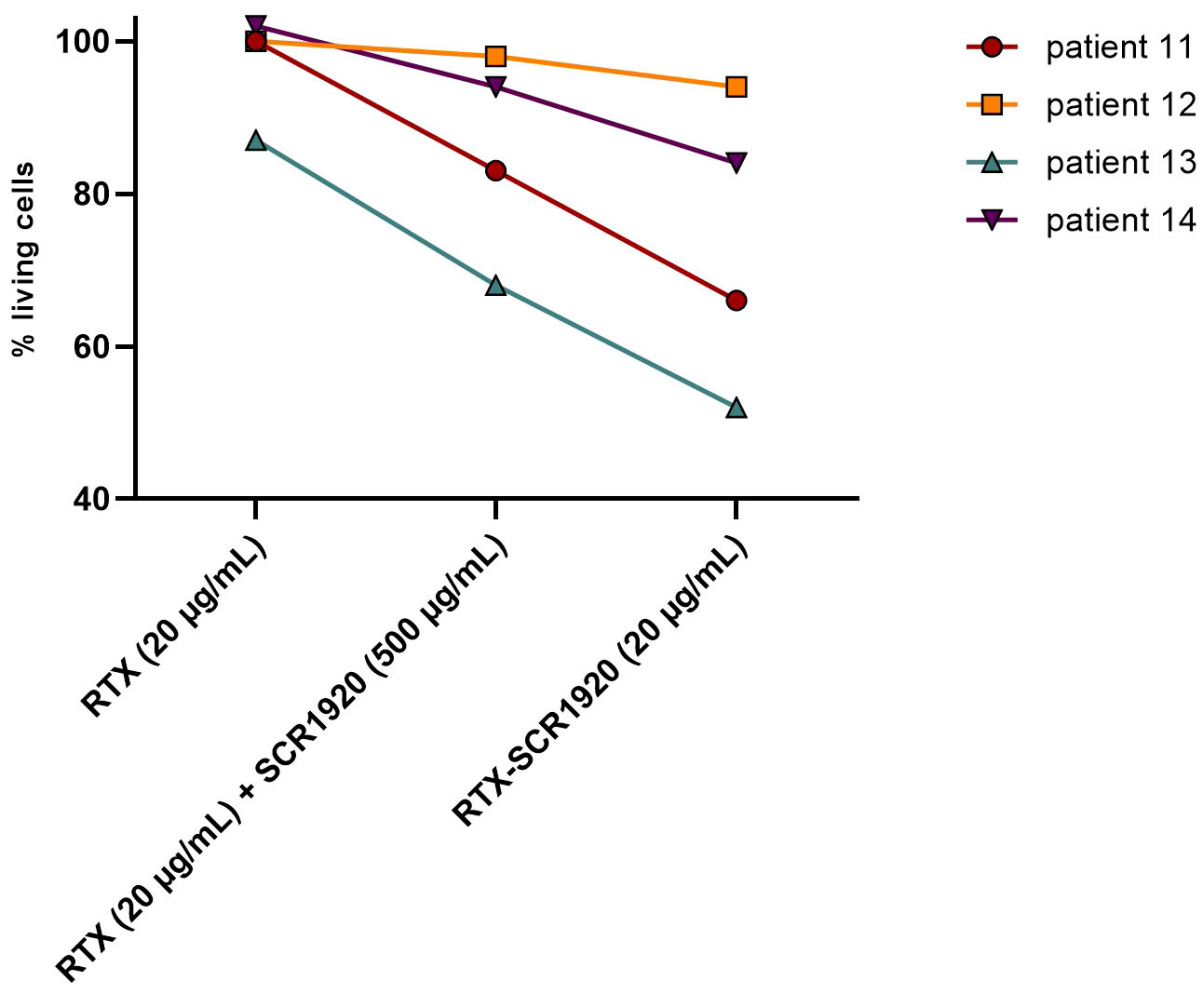
CLL lysis assay on 2x10^5^ PBMCs/sample of 4 different CLL patients (patient IDs 11, 12, 13, 14), comparing parental RTX (20 µg/mL), a combination of RTX (20 µg/mL) and SCR1920 (500 µg/mL) and RTX-SCR1920 (20 µg/mL). Twenty percent NHS were added as complement source. The median is shown (n=3). Percentages of living cells were calculated as a ratio of viable cells in a hiNHS containing control sample.

First, parental RTX, RTX combined with SCR1920 peptide and the fusion RTX-SCR1920 were compared side by side in a lysis test on the PBMCs of four different CLL patients. Antibodies were used at a concentration of 20 µg/mL, SCR1920 peptide at 500 µg/mL, which corresponds to a 10-fold molar excess compared to the physiological concentration of fH. The responses to the treatment among the four patients were highly variable but overall demonstrated a similar pattern. The RTX single treatment had no or poor effects in the selected patients, which were therefore classified as CDC-non-responders with less than 15% lysis induction when compared to the hiNHS samples (9, 12). When RTX treatment was concomitantly administered with SCR1920 treatment, a decrease in the proportion of living cells ranging from 8 to 19% decrease was evident (**Figure 3**). Only in patient 12 there was a poor response to the SCR addition. With the administration of the RTX-SCR1920 fusion-construct, the efficacy of RTX-induced lysis could further be enhanced by 10 to 17% compared to the combinational treatment with RTX and SCR1920 peptide. Notably, even in patient 12, who showed neither response to the OFA nor to the combination of OFA and the SCR1920 peptide, a minor reduction of viable cells was recorded.

**Figure 4:**
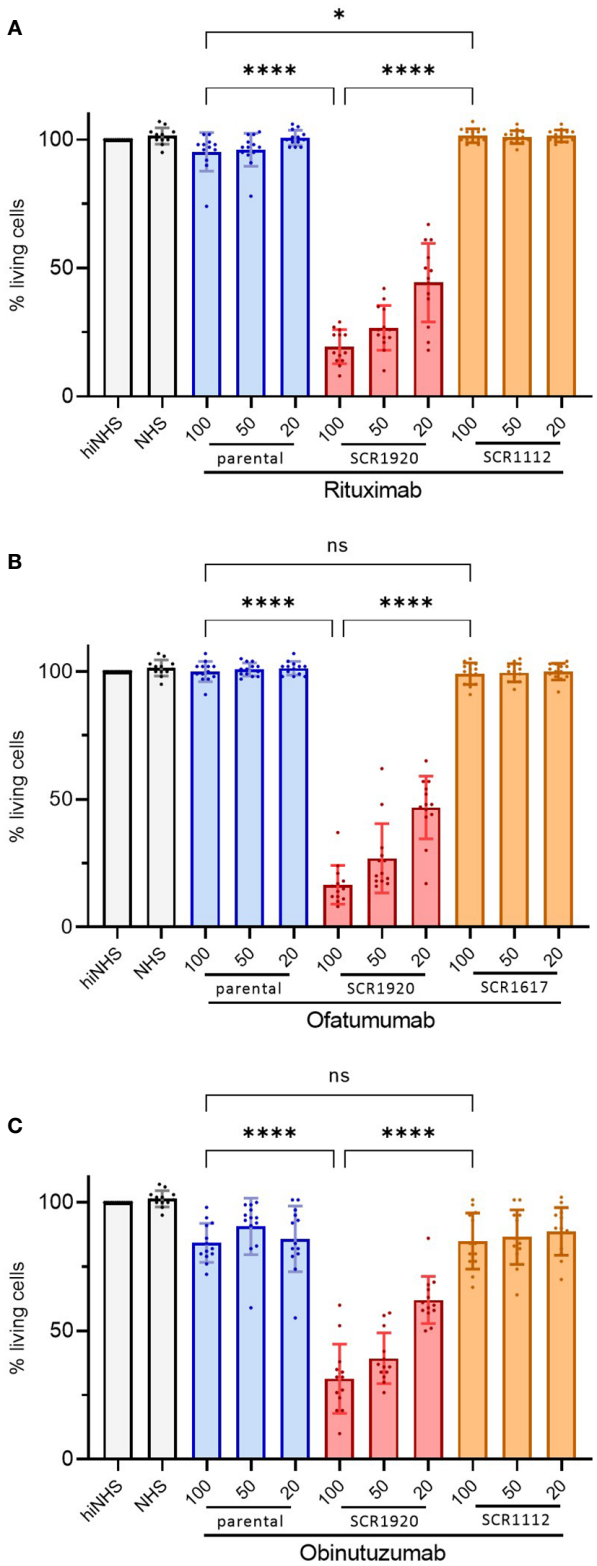
SCR1920 increases complement-dependent cytotoxicity of RTX **(A)**, OBI **(B)** and OFA **(C)** in patient samples, not responding to the parental antibody, significantly. 2x10^5^ CLL patient’s PBMCs were incubated with 20, 50 or 100 µg/mL mAb and 20% NHS for 1 h at 37°C. The proportion of PI-negative, living cells was determined by flow-cytometry (60 sec acquisition, high flow rate), relative to a hiNHS containing control sample. The means and standard deviations of 4 patients, each tested in 3 individual assays are shown. One-way ANOVA and Tukey’s multiple comparisons test were performed. *p<0.05, ****p<0.0001; ns, not significant; mean+/-SD.

As these results highlighted the increased lysis potential of SCR1920 conjugated RTX, this finding was validated in a larger experiment comparing parental, SCR1920 and SCR1112/SCR1617 fusion-constructs of RTX, OFA and OBI side by side on PBMCs of CD20 mAb treatment naïve CLL patients (**Figures 4A–C**). In total, four CDC-non-responders were tested in three individually repeated experiments. The cell survival upon antibody treatment was calculated relatively to a control sample without antibody treatment but containing 20% hiNHS. All other samples contained 20% NHS as a source for complement. Statistical significance was calculated by one-way ANOVA and Tukey’s multiple comparisons test (*p<0.05 ****p<0.0001). Patients chosen for this study did not respond to the treatment with the parental mAbs *in vitro*. Likewise, there was no or only a minor difference between the parental and the SCR1112/SCR1617-fused controls, excluding an effect of the increased mAb size *per se*. However, the SCR1920 fusion-constructs increased the lysis capacity of all three tested mAbs significantly compared to the parental and the SCR1112/SCR1617-fused control Abs, reaching levels of less than 20% living cells in the highest doses of RTX-SCR1920 and OFA-SCR1920 and almost 25% remaining viable cells in the highest dose of OBI-SCR1920. A concentration-dependent effect was observed. The variability between patients was relatively high but still, a very significant enhancement of lysis capacity by SCR1920 fusion-constructs was detected, when calculating the mean and SD of all measurements. Overall, the utilization of CD20 mAb-SCR1920 fusion-constructs exhibited the capacity to transform patients who initially displayed non-responsive behavior to antibody treatment *in vitro* into treatment responders. These results underscore the potential of the mAb-SCR1920 fusion-technology for the improvement of mAb-based therapeutics.

### 3.4 Lysis induced by mAb-SCR1920 constructs was specifically restricted to B cells

**Figure 5:**
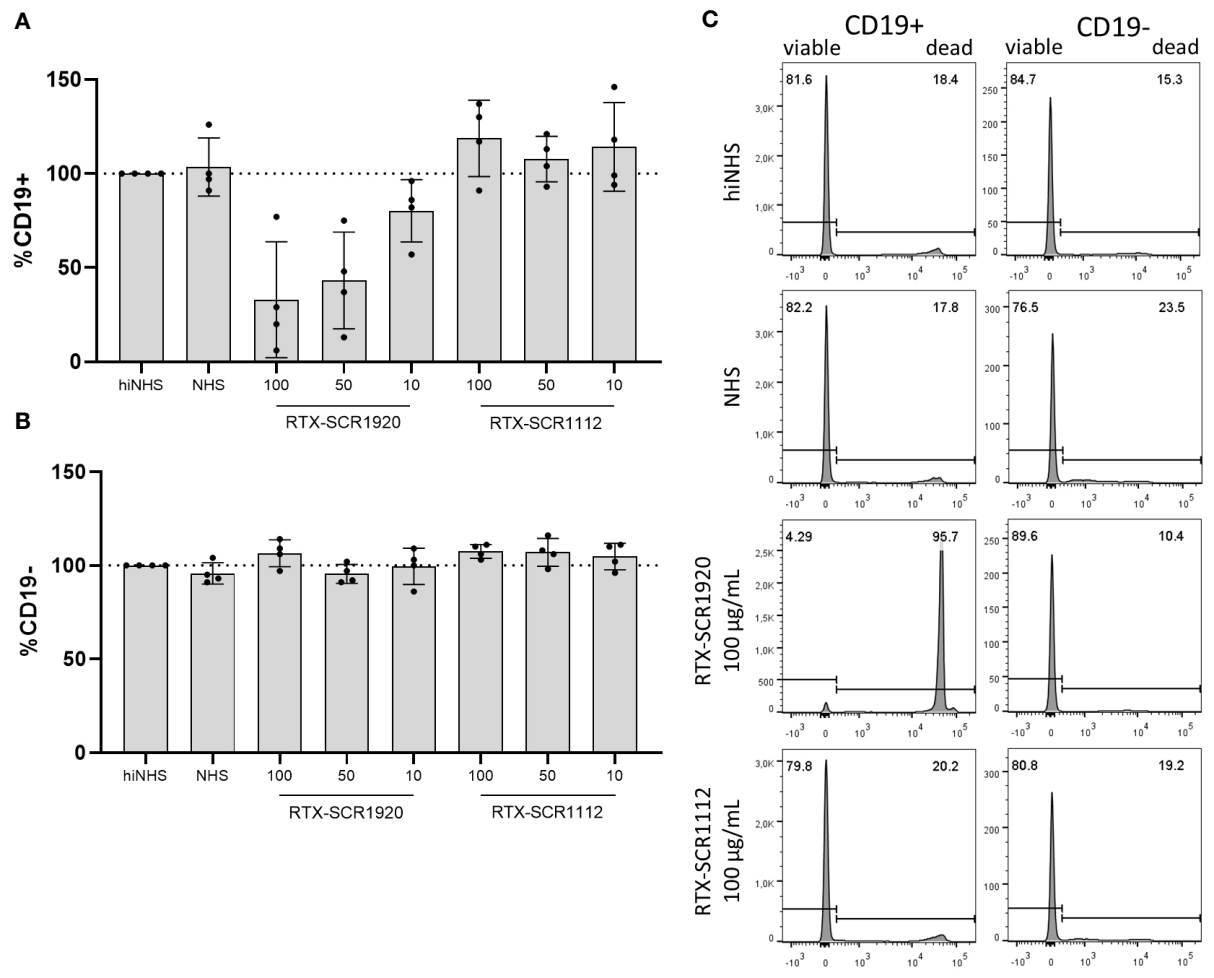
Lysis induced by RTX-SCR1920 is restricted to CD19-positive cells and does not affect CD19-negative cell populations significantly. PBMCs from four CLL patients were treated with RTX-SCR-constructs in concentrations of 100, 50 or 10 µg/mL in a CDC assay. CD19+ **(A)** and CD19- **(B)** cells were stained and analyzed by flow-cytometry. Survival rates (PI-negative cells) were calculated relative to hiNHS sample. n=4; mean+/-SD **(C)** The treatment of PBMCs with RTX-SCR1920 led to a massive increase in PI-positive, dead CD19-expressing cells. This effect did not appear with the RTX-SCR1112 antibody. In contrast, a decrease of PI-negative living cells was not visible in CD19-negative populations. The results of one representative measurement are shown.

The fusion of SCR1920 to mAbs should increase CDC specifically on target cells of the antibody that is conjugated to the SCR. In order to validate this target specificity, CLL PBMCs were treated with RTX-SCR fusion-constructs in a standard CDC assay. After staining with APC-conjugated anti-human CD19 antibody, cells were analyzed by flow-cytometry. Across the four patients tested, there was no response to the RTX-SCR1112 treatment. However, lysis was notably augmented with the application of 100 µg/mL RTX-SCR1920, resulting in 33% viable cells on average (**Figure 5A**). The percentages of CD19-negative cells (**Figure 5B**) were not significantly influenced by the treatment with the different antibody constructs and concentrations (**Figure 5C**). Further, the presence of complement C3c on CD19-negative cells was assessed. Independent on the modification of the mAbs, an increase of C3c deposition was observed, which was around two-times when compared to the NHS control without mAb in the experimental setting. No significant increase in C3c levels on cell surfaces was observed, when examining SCR1920-conjugated mAbs in comparison to their parental counterparts (data not shown).

### 3.5 The addition of soluble or linked SCR1920 to OFA increased CDC on low and high level CD20 expressing B cells

The correlation between CD20 expression on CLL cells from eight distinct patients and their responsiveness to OFA-SCR1920 was investigated as it has been shown to correlate with the CDC efficacy of CD20 mAbs like RTX (9). CD20 expression levels in the individual patient samples were determined by FACS analysis using OFA (20 µg/mL) followed by the detection of bound OFA with an anti-human-IgG FITC-labeled Ab. Simultaneously, PBMCs of the patients were incubated with OFA (20 µg/mL), OFA (20 µg/mL) in combination with soluble SCR1920 peptide (1.2 mg/mL) or OFA-SCR1920 fusion-construct (20 µg/mL) in the presence of NHS as a source of complement.

**Figure 6:**
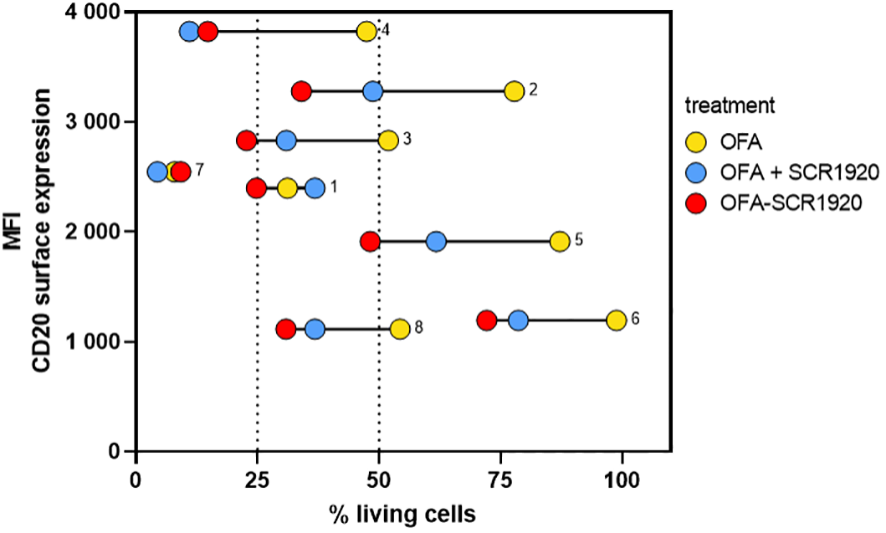
SCR1920 can increase CDC of OFA on low and high level CD20 expressing B cells. CLL PBMCs from 8 different patients were incubated for 1 h with 20 µg/mL of OFA, OFA with 1,200 µg/mL SCR1920 or OFA-SCR1920. Twenty percent NHS was added as source for complement. PI-negative, viable cells were counted by flow-cytometry for 60 sec at a constant flow rate. Survival rates were calculated relative to a hiNHS sample. In parallel, CD20 expression of patients was determined by incubating CLL PBMCs of each patient with 5 µg/mL OFA for 30 min at 4°C. After washing, OFA bound cells were detected with an anti-human-IgG-FITC antibody. Ten thousand events were recorded to determine the mean fluorescence intensity (MFI) of FITC.

The MFI of CD20 expression versus percentages of living cells were juxtaposed. Among patients treated with the parental OFA, five tested individuals displayed CLL cell survival rates exceeding 50% and only three of them (patients #1, 4, 7) exhibited higher susceptibility. The combinations of OFA with soluble SCR1920 peptide and the fusion OFA-SCR1920 construct both demonstrated an ability to increase responsiveness. When combining OFA with SCR1920 peptide, a clear shift toward decreased cell survival rates was evident, indicating an enhanced eradication of CLL cells. This effect was further amplified for the fused OFA-SCR1920 antibody. In this group, cells from four patients were lysed with an efficacy of less than 25% surviving cells and only one (patient 6) remained with lysis induction above 50% (**Figure 6**).

### 3.6 Increased lysis capacity of antibody-SCR conjugates mainly relies on CDC rather than ADCC or phagocytosis

As complement activation and the deposition of C3-fragments might affect ADCC, we tested the effect of the presence of NK cells on the clearance of CLL cells induced by mAb-SCR constructs *in vitro* (13).

**Figure 7:**
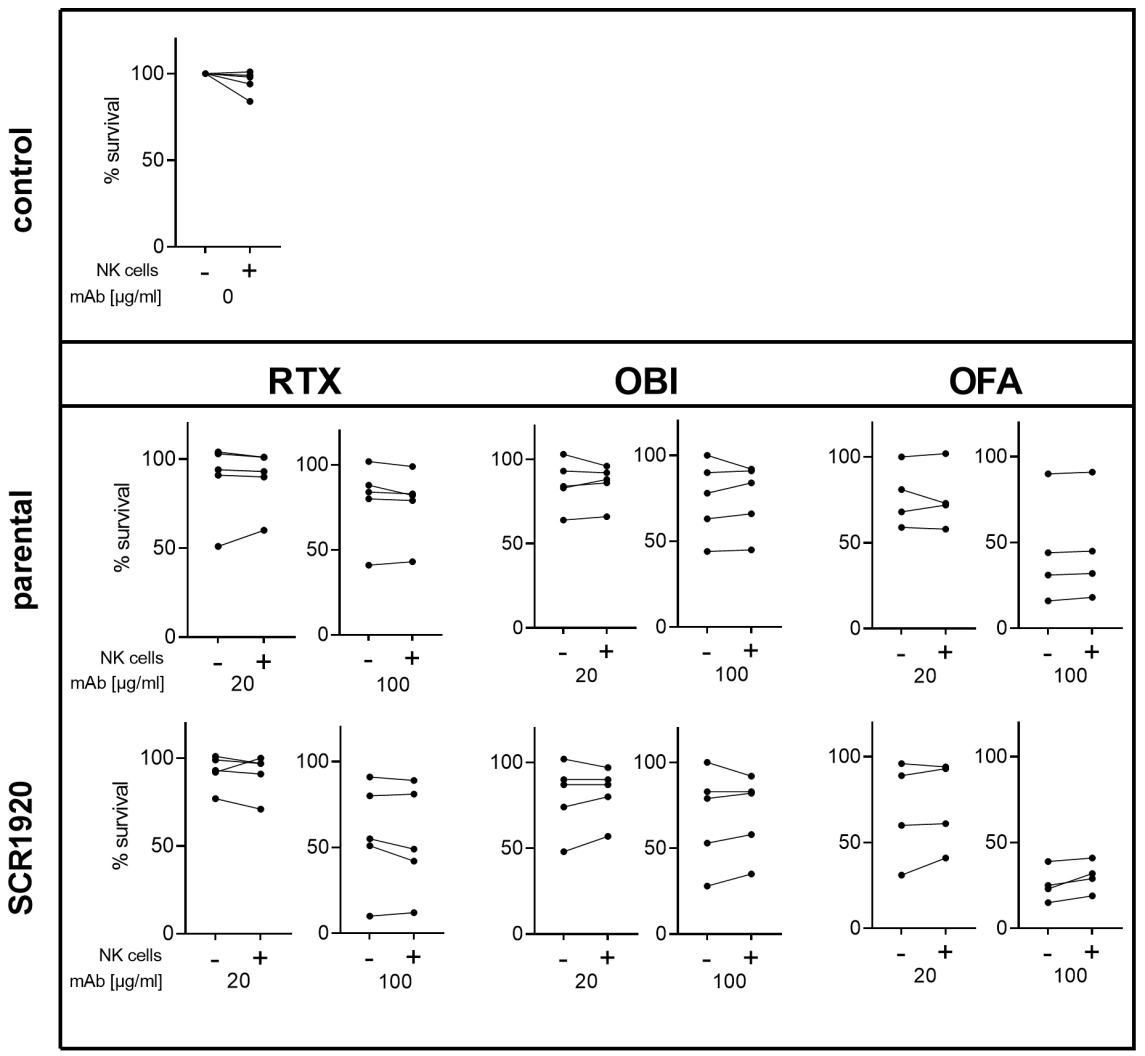
The presence of NK cells in patient’s PBMCs does not hinder increased CDC activation by SCR1920 conjugated mAbs. The fraction of surviving PBMCs after treatment with parental or SCR1920-conjugated RTX, OBI and OFA of five different CLL patients is shown. Samples were split, half was left in its original composition (indicated by “+”), and the other half was depleted of NK cells (indicated by “-”). Standard CDC lysis tests were performed with 20% NHS as a source for complement. Values were normalized to a hiNHS containing control sample and the percentages of surviving cells were calculated. Results for each individual patient are connected with a line. n(RTX, OBI)=5, n(OFA)=4.

Five randomly selected CLL patients were included in this experiment, with each patient sample divided into two portions. In one half, NK cells were removed from PBMCs (**Supplementary Figure 4B**), while the second half was left in its original cellular composition (**Supplementary Figure 4A**). Both cell fractions were tested in standard complement lysis assays. Percentages of viable cells were calculated relative to a hiNHS containing control sample. Antibodies were tested at 20 and 100 µg/mL. A concentration-dependent lysis enhancement was measurable for all three SCR1920 conjugated mAbs (**Figure 7**). Additionally, an increase in lysis capacity was observed for all three mAbs upon comparison of parental constructs with the SCR1920-conjugated counterparts. When comparing the means of NK cell depleted and preserved samples, no significant differences were measurable across any antibody treatment (mean and SD data are not depicted here). Hence, we could conclude that the increased lysis capacity of SCR1920-conjugated antibodies is predominantly driven by increased CDC rather than relying on ADCC and the presence of NK cells. It should be noted that the OBI used in these experiments were fucosylated which may explain the poor ADCC response in this setting.

Further, the effect of the SCR-conjugation on ADCC in the presence of different effector to target (E:T) ratios was assessed. Overall, the SCR1920-conjugation was able to decrease the viability of target cells in the presence of NHS or hiNHS at both tested E:T ratios (10:1 and 5:1) at mAb concentrations of 10 µg/mL or higher and was independent on the modification (**Supplementary Figure 5**).

Additionally, it was assessed if phagocytosis as an antibody effector mechanism is affected by the antibody-SCR1920 fusion protein compared to its parental counterpart. Phagocytosis was inhibited by adding 1 µM of spleen tyrosine kinase (SYK) inhibitor BI-1002494 and lysis capacity of antibodies was tested in a standard CLL lysis assay on five CLL patients. Overall the stronger lytic effect of SCR1920-coupled antibody was maintained even with the addition of the SYK inhibitor. The differences between the samples were not significant and the inhibition of phagocytosis by blocking SYK did not seem to have an effect on the lysis capacity of the SCR-antibody constructs (**Supplementary Figure 6**).

## 4 Discussion

This study aimed to establish a complement factor H-SCR based system to improve the lysis efficacy of mAbs for the depletion of certain cell populations exemplified by CLL cells. Monoclonal Ab-SCR fusion-constructs were *in vitro* produced and purified by affinity chromatography.

*In vitro* CLL lysis assays indeed confirmed the superior dose-dependent and target cell specific CDC activity achieved by fusion of SCR1920 with the CD20 mAbs RTX, OFA and OBI. This finding is mechanistically based on the displacement of full-length fH from mAb target cells by SCR1920-tails (9, 10). This might be due to the interference of SCR1920 with both, the binding of fH to glycosaminoglycans or the interaction of fH with C3b, deposited on the cell surface after complement activation. The complement inhibiting function of full-length fH, which is located in SCRs1–4, is eliminated, when SCR1920 is present. By removing fH, the cells are sensitized for the activation of complement. SCR1112- or SCR1617-constructs were chosen as controls which should not alter CDC of mAbs because they do not harbor known domains involved in the binding to cell surface structures or complement regulation (7). Owing to the high concentrations necessary to impair fH binding, the direct application of SCR1920 is not feasible *in vivo*. With serum concentration of fH ranging from 0.235 to 0.81 mg/mL, a direct inhibition of fH by mAbs against fH may also be hampered by high amounts of Ab needed to be effective. Thus, SCR1920 has to be coupled directly to anti-CD20-mAbs, which may provide several advantages: (i) the low-affinity peptide would be shuttled by anti-CD20-mAbs specifically to CD20-expressing cells and compete with fH directly on the spot; (ii) due to the law of mass action, lower amounts of SCR are needed when the SCR is directed preferentially to CLL cells by the high affinity of the therapeutic Ab; (iii) when binding to non-B cells via the SCRs, serum fH will immediately remove the SCR-mAb construct, as the anti-CD20-Abs-SCR is in the nanogram range and thus in much lower concentration in the system compared to fH; (iv) owing to the increased efficacy, such a bifunctional anti-CD20-mAbs-SCR molecule may turn patients susceptible to the Ab therapy, which are refractory to anti-CD20-mAbs treatment. The feasibility of such a strategy has only recently been demonstrated by our group showing an improved complement-mediated virolysis when SCR18–20 was linked to a virus-specific Ab (14). A similar strategy was also previously utilized to target HER2-positive cancer cells using fH-related protein 4-based immunoconjugates (15).

The use of primary patient material for the efficacy testing of our antibody constructs had the advantage of displaying inter-patient variations in the treatment response and the natural presence of different cell types within the PBMC pool. On the other hand, this variety of patients with different disease burden and staging as well as pre-treatment or expression levels of CD20, all of which may affect the treatment response, impeded the finding of clear conclusions. However, there was a tendency that lysis of CLL cells correlated with the expression of CD20 on the cell surface (9, 13, 14). This relatively low level of correlation might be due to the presence of additional complement regulators such as CD55 or CD59 (9, 10, 16). In order to demonstrate that the fusion of parental mAbs with fH-derived SCR1920 improved the efficacy of the mAbs, patients, not responding to the *in vitro* treatment with parental antibodies were included in the mAb-SCR lysis study.

It has to be mentioned that the OBI used here was not in its core-afucosylated state as it is in its commercial version (17). The commercial production of afucosylated OBI is performed in a CHO cell line, overexpressing the β-1,4-N-acetylgluosaminyltransferase III. The overexpression of this enzyme leads to the downstream inhibition of the α-1,6,fucosyltransferase, which causes the enrichment of bisecting oligosaccharides rather than fucosylation (18). Independent on fucosylation, the Fc-portion of the Ab should be able to interact with C1q (19, 20). However, C1q binding and subsequent complement activation, sufficient to disrupt the integrity of the cellular membrane, is only observed at high OBI concentrations, due to the impaired translocation of CD20 into lipid rafts by this mAb (3). Thus, we were taken by surprise that the conjugation of OBI with SCR1920 induced complement-mediated lysis already at relatively low Ab concentrations.

The interplay of ADCC and CDC as effector mechanisms of mAbs is still discussed controversially. On one hand, it is described that antibody-mediated lysis induced by RTX and OBI can be significantly increased when complement is restricted or depleted (13, 21, 22). For example, mAbs that are low in fucose were found to exhibit increased FcγRIII-binding capabilities and thus induce enhanced levels of ADCC (18). On the other hand, it was shown that CDC and ADCC of mAbs can also be enhanced simultaneously by Fc protein- and glyco-engineering approaches (23). Our results emphasize the superiority of SCR1920-conjugated antibodies compared to their parental counterparts when assessing CDC as well as ADCC.

Several studies found increased counts of NK cells in CLL patients but no correlations between NK counts and clinical staging or known prognostic factors of the disease were revealed (24, 25). Patients with a higher ratio of NK cells to CLL cells seem to have a longer time to treatment-onset, according to one study (26). Another study found a correlation between absolute NK counts and overall survival (27). Interestingly, ADCC response on mAbs by NK cells is also influenced by genetic factors. The clinical response rate to RTX was found to be significantly higher in patients, homozygous for the FcγRIIIA-158F allele (28).

An overall NK cell dysfunction with decreased cytotoxicity has also been observed repeatedly in CLL patients (24). The co-culture of NK cells with CLL cells or the incubation of NK cells with plasma from CLL patients were shown to inhibit the cytotoxic function of NK cells *in vitro* (29–31). The presence of impaired NK cells and the fact that RTX and OFA treatment were found to induce a decrease of CD16 expression and subsequent diminished ADCC function of NK cells further emphasizes the promising potential of mAb-SCR1920 constructs with increased CDC activity (24).

In future studies, the effects of OBI-SCR1920 defucosylation should further be explored to gain more understanding of the complex interplay between CDC and ADCC especially in the field of glycoengineered mAbs. The combination of defucosylated OBI with SCR1920 may additionally improve the anti-clonal activity of CD20-positive lymphoma cells, including CLL, as OBI has shown superiority in a clinical side-by-side comparison with RTX (32). However, despite improved OBI-based combination therapies, such as venetoclax/OBI, many patients remain on a significant minimal residual disease positivity level (>10^-4^), which has been linked to inferior outcome. Thus improved engineered mAbs would be an attractive tool to increase therapeutic efficacy.

Overall, our study emphasizes the crucial role of CDC in the elimination of target cells by mAbs *in vitro* and introduces a novel technology for enhancing mAb efficacy independent from the presence of NK cells through the fusion of fH SCR1920 to mAbs. These findings set the stage for a platform systemically improving effector mechanisms of various mAb-based therapeutics by boosting mAb-triggered CDC induction.

## Data availability statement

The original contributions presented in the study are included in the article/**Supplementary Material**. Further inquiries can be directed to the corresponding author.

## Ethics statement

This study was approved by the Ethics Committee of the Medical University of Innsbruck (approval no. 1154/2019). After receiving informed consent, peripheral blood mononuclear cells (PBMCs) were obtained from heparinized blood of anti-CD20 antibody therapy naïve CLL patients at the University Clinics Innsbruck, Department of Internal Medicine V, Hematology & Oncology. The studies were conducted in accordance with the local legislation and institutional requirements. The participants provided their written informed consent to participate in this study.

## Author contributions

LP: Writing – original draft, Writing – review & editing, Investigation, Methodology, Validation, Visualization. PH: Investigation, Methodology, Writing – review & editing. LB: Investigation, Methodology, Writing – review & editing. KC: Writing – review & editing, Methodology, Investigation, Data curation. J-PB: Investigation, Writing – review & editing, Resources. DW: Investigation, Resources, Methodology, Writing – original draft. ZB: Investigation, Methodology, Formal analysis, Supervision, Validation, Writing – review & editing. AB: Methodology, Writing – review & editing, Resources. MP: Methodology, Resources, Writing – review & editing. GH: Methodology, Resources, Writing – review & editing. SS: Resources, Writing – review & editing. CT: Resources, Investigation, Methodology, Project administration, Writing – original draft. HS: Writing – original draft, Conceptualization, Writing – review & editing.

## Supplementary material

Supplementary Figure 1 DLS analyses of RTX-SCR1920 **(A)** and OBI-SCR1920 **(B)**, formulated differently: (3, blue) unformulated (elution buffer Tris-Gly-HCl); (2, red) elution buffer with 1:1,200 Trehalose, 10 mM L-His, 0.05% (w/v) Kolliphor P188; (1, green) elution buffer with 1:1,200 Trehalose, 20 mM L-His, 0.05% (w/v) Kolliphor P188. The average signal (line) and the standard deviation (shading) are shown. Measurement of hydrodynamic radius of samples by DLS. The average signals (lines) with the standard deviations (shades) are given.

Supplementary Figure 2 NanoDSF analyses of RTX-SCR1920 **(A)** and OBI-SCR1920 **(B)**, formulated differently: (3, blue) unformulated (elution buffer Tris-Gly-HCl); (2, red) elution buffer with 1:1,200 Trehalose, 10 mM L-His, 0.05% (w/v) Kolliphor P188; (1, green) elution buffer with 1:1,200 Trehalose, 20 mM L-His, 0.05% (w/v) Kolliphor P188. The average signal (line) and the standard deviation (shading) are shown. The macroaggregation (turbidity) and thermal melting were analyzed at increasing temperatures by light scattering and absorption measurements, respectively. T-IP, solid vertical line, representing the temperature of the inflection points determined from the first derivative of the thermal melting curve; T-ON, dashed vertical line, temperature at which the onset of macroaggregation was determined by light back-scatter;.

Supplementary Figure 3 The CD20 binding capacity of clinically used RTX, RTX in-house, RTX-SCR1920 and RTX-SCR1112 does not differ significantly. 2x10^5^ PBMCs of 3 different CLL patients were stained with 5 µg of the respective RTX-variant. Surface-bound RTX was detected with an anti-human-IgG-FITC-conjugated secondary antibody. The mean fluorescence intensity (MFI) of the positive population was determined and compared by performing ordinary one-way ANOVA and Tukey’s multiple comparison test. ns, not significant; background, only secondary antibody; n=3; means +/-SD are shown;.

Supplementary Figure 4 The efficacy of NK cell depletion was confirmed by flow-cytometry. PBMCs of included CLL patients were analyzed pre **(A)** and post **(B)** *in vitro* NK cell depletion for the presence of CD16+CD56+ NK cells.

Supplementary Figure 5 OBI-SCR1920 induces increased levels of cell killing when different effector:target (E:T) ratios were tested. NK cells isolated from healthy donor blood were combined with 2x10^5^ MEC-1 cells at E:T ratios of 1:5 and 1:10. Standard CLL lysis assays with OBI or OBI-SCR1920 at concentrations ranging from 1–100 µg/mL and 20% NHS or hiNHS were performed. Viable cells were determined by PI staining and flow-cytometry. Values were normalized to a hiNHS containing control sample. n=2; means +/-SD are shown;.

Supplementary Figure 6 Blocking phagocytosis by inhibiting the spleen tyrosine kinase (SYK) does not have an effect on the overall lysis capacity of antibody-SCR1920 conjugates. PBMCs from CLL patients were depleted of NK cells and 2x10^5^ cells per sample were analyzed in a standard CLL lysis assay with 20% NHS and 50 µg/mL of parental or SCR1920-conjugated RTX **(A)**, OBI **(B)** or OFA **(C)** in combination with or without 1 µM SYK. Viable cells were determined by PI staining and flow-cytometry. Values were normalized to a NHS containing control sample. n=5; means +/- SD are shown.
