# Supplementary material for: Enhancement of complement-dependent cytotoxicity by linking factor-H derived short consensus repeats 19-20 to CD20 antibodies
Source: Front Immunol. 2024 Jul 22;15:1379023. doi: 10.3389/fimmu.2024.1379023 (PMC11298693; doi:10.3389/fimmu.2024.1379023)
Supplement: Supplementary Figure 1 — DLS analyses of RTX-SCR1920 (A) and OBI-SCR1920 (B), formulated differently: (3, blue) unformulated (elution buffer Tris-Gly-HCl); (2, red) elution buffer with 1:1,200 Trehalose, 10 mM L-His, 0.05% (w/v) Kolliphor P188; (1, green) elution buffer with 1:1,200 Trehalose, 20 mM L-His, 0.05% (w/v) Kolliphor P188. The average signal (line) and the standard deviation (shading) are shown. Measurement of hydrodynamic radius of samples by DLS. The average signals (lines) with the standard deviations (shades) are given. [file DataSheet_1.pdf]

Supplementary Material

Determination of purity and thermal stability of mAb-SCR by DLS and nanoDSF

Purity and thermal stability of in-house produced mAb-SCR constructs were assessed by 2bind GmbH, Regensburg, Germany. For this, dynamic light scattering (DLS) and nano-differential scanning fluorimetry (nanoDSF) were performed. In brief, a Prometheus Panta device (NanoTemper Technologies) was used to perform a multi-parameter stability characterization of mAb-SCR samples. DLS experiments for size analysis were performed at 20°C. To determine folding and aggregation parameters by nanoDSF samples were heated up from 20°C to 95°C with a heating speed of 1°C/min. Thermal protein unfolding was measured by a spectral shift in the fluorescence emission profile of the tryptophan and tyrosine residues, measured at 350 nm. The macroaggregation of the target protein was monitored by light backscatter using the Prometheus NT.48 back-reflection optics.

In-house produced and purified antibodies were assessed for their quality and stability by DLS and nanoDSF. RTX-SCR1920 (**Suppl**. **Fig.1A**) and OBI-SCR1920 (**Suppl**. **Fig.1B**) were tested in three different buffer conditions. The unformulated sample in Tris-Gly-HCl elution buffer (pH 6.5; **blue lines in Suppl. Fig. 1**) was compared against a sample in elution buffer with 1:1,200 Trehalose, 10 mM L-His, 0.05% (w/v) Kolliphor P188 (**red lines**) or as a third option elution buffer with 1:1,200 Trehalose, 20 mM L-His, 0.05% (w/v) Kolliphor P188 (**green lines**). DLS analyses showed that in-house produced antibodies were of acceptable and sufficient purity. This was indicated by the sharp peaks of DLS measurement curves. Mean hydrodynamic radii between 5.93 to 6.97 nm for RTX-SCR1920 samples (**Suppl**. **Fig.1A**) and 6.00 to 6.36 nm for OBI-SCR1920 samples (**Suppl**. **Fig. 1B**) were measured. Additional species between 100 to 10,000 nm were observed for both antibodies in all three formulation buffers. These higher molecular weight species were of low abundance and therefore neglectable.

Further, the turbidity signal determined by nanoDSF back-scatter measurements at rising temperatures served as indicator for microaggregation of the sample. One aggregation transition was observed between around 70°C and 80°C for all tested samples (**Suppl**. **Fig. 2A**). NanoDSF results showed the typical biphasic thermal melting transition of antibodies with two major unfolding events that corresponded most likely to the unfolding of individual antibody domains. One unfolding event for the C_H2 domain and the second for the C_H3 and Fab domains (see subgraphs "Thermal melting, first derivative" in the **Suppl. Fig. 2A**).

The thermal inflection points (T-IP) represent the temperatures at which approximately 50% of the sample/domain are unfolded. For RTX-SCR1920 two unfolding transitions were observed between 73.48-

73.78°C and 78.57-80.73°C (**Suppl**. **Fig. 2A**). For OBI-SCR1920 one broad unfolding transition was observed between 76.40-76.51°C for samples 1 and 2 and two unfolding transitions were observed for sample 3 (74.45°C and 79.41°C; (**Suppl**. **Fig. 2B**)). This difference in sample 3 was associated to the higher concentration of this sample. The signal-to-noise level of all unfolding transitions sufficiently low and the unfolding transition temperatures could be determined with high accuracy. The unfolding inflection point values were similar for the three tested buffer conditions.

Antigen binding capacity of SCR-fusion constructs is not impaired compared to the parental antibody

The CD20 binding capacity of RTX-SCR constructs was tested on $2x10^5$ CLL PBMCs of three different patients. The samples were treated with 5 µg RTX clinic, RTX in-house, RTX-SCR1920 or RTX-SCR1112 and incubated for 30 min at 4°C. After washing, the surface bound RTX was detected with a polyclonal rabbit anti-human IgG-FITC (Agilent Technologies) conjugated secondary antibody (incubation 30 min, 4°C). The mean fluorescence intensity (MFI) of the FITC-positive population was determined by flow-cytometry.

As patients differ in their CD20 surface expression, the MFIs varied between the three tested PBMC samples. The mean and SD were calculated and the statistical significance was analyzed by ordinary one-way ANOVA and a subsequent Tukey's multiple comparisons test (**Suppl. Fig. 3**). The antigen binding capacity did not differ significantly between the clinically used RTX, the in-house produced RTX, RTX-SCR1920 and RTX-SCR1112. Therefore, different binding capacities can be excluded as a reason for different target cell lysis capacities.

Verification of *in vitro* NK cell depletion in CLL PBMCs by flow-cytometry

The depletion of NK cells from CLL PBMCs was confirmed by staining PBMCs pre and post depletion with BD multitest CD3 FITC / CD16 PE + CD56 PE / CD45 PerCP / CD19 APC according to manufacturer's recommendations (BD Biosciences).

CD16+CD56+ PE-stained cells were quantified and compared. **Suppl. Fig. 4** shows a representative sample of a patient. The proportion of NK cells decreased from 11.3% of the overall lymphocyte count before treatment to 0.52% after NK depletion, corresponding to a decreased NK cell count of approximately 95%.

ADCC assay

To evaluate ADCC induced by mAb-constructs, $2x10^5$ MEC-1 cells (Leibniz Institute, DSMZ, Braunschweig, Germany) were combined with NK cells isolated from healthy donors in effector to target ratios of 1:5 or 1:10. Ab-constructs were added at 1-100 µg/mL in combination with 20% NHS or hiNHS. The samples were incubated at 37°C, 5% CO_2 for 4 h and the amount of viable cells was determined by PI staining and flow cytometry as described above (**Suppl. Fig. 5**). Significances of changes induced through different E:T ratios were determined by two-way ANOVA and Tukey's multiple comparisons test. Significances were not included in **Suppl. Fig. 5** for visibility reasons. The differences between means of OBI and OBI-SCR1920 treated cells with E:T ratios of 1:5 or 1:10 was only significant at 100 µg/mL antibody (*p=0.0172; ** p=0.0015 respectively). For the hiNHS setting, only samples containing 50 and 100 µg/mL antibody and an E:T ratio of 1:5 displayed a significant difference when comparing OBI and OBI-SCR1920 treated samples (**p=0.0089; *p=0.0298 respectively).

Phagocytosis inhibition assay

Spleen tyrosine kinase (SYK) inhibitor BI-1002494 (Boehringer Ingelheim, Ingelheim am Rhein, Germany) was used to inhibit phagocytosis exerted by macrophages and neutrophils. As suggested by the manufacturer, 1 µM of the inhibitor was added to each sample of a standard CLL lysis assay (as described in the main part) including 5 randomly chosen CLL patients. The patients' PBMCs were depleted from NK cells using the REAlease CD56 MicroBead Kit (Miltenyi Biotec), as described earlier to exclude that measured effects rely on ADCC rather than phagocytosis and CDC. All samples were normalized to a control sample, without antibody treatment or inhibitor, containing only NHS. The parental antibodies and SCR1920-conjugated constructs of RTX, OBI and OFA were tested at 60 µg/mL with 1 µM SYK ("+SYK-NK"), without SYK ("-SYK-NK") or without SYK but with NK cells (= PBMC composition prior to NK depletion; "-SYK+NK").

A

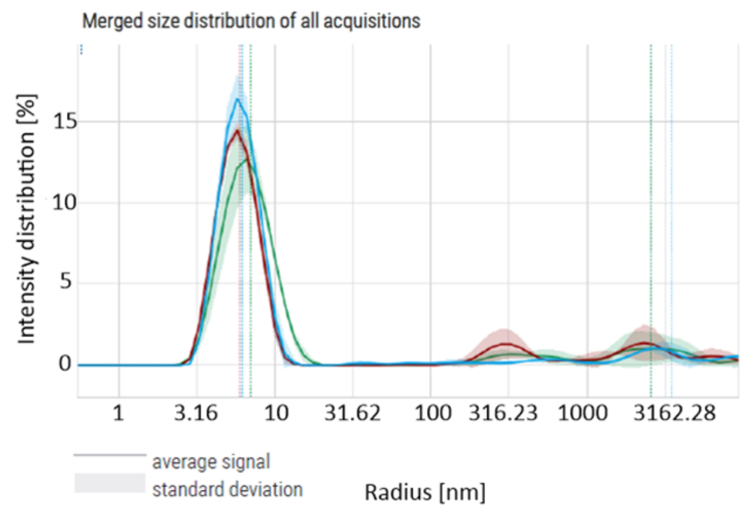

B

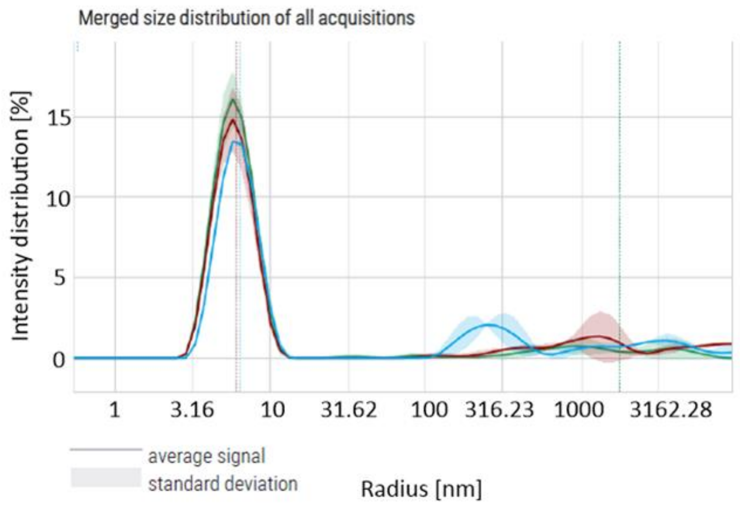

Suppl. Figure 1: **DLS analyses of RTX-SCR1920 (A) and OBI-SCR1920 (B), formulated differently**: (3, blue) unformulated (elution buffer Tris-Gly-HCl); (2, red) elution buffer with 1:1,200 Trehalose, 10 mM L-His, 0.05% (w/v) Kolliphor P188; (1, green) elution buffer with 1:1,200 Trehalose, 20 mM L-His, 0.05% (w/v) Kolliphor P188. The average signal (line) and the standard deviation (shading) are shown. Measurement of hydrodynamic radius of samples by DLS. The average signals (lines) with the standard deviations (shades) are given.

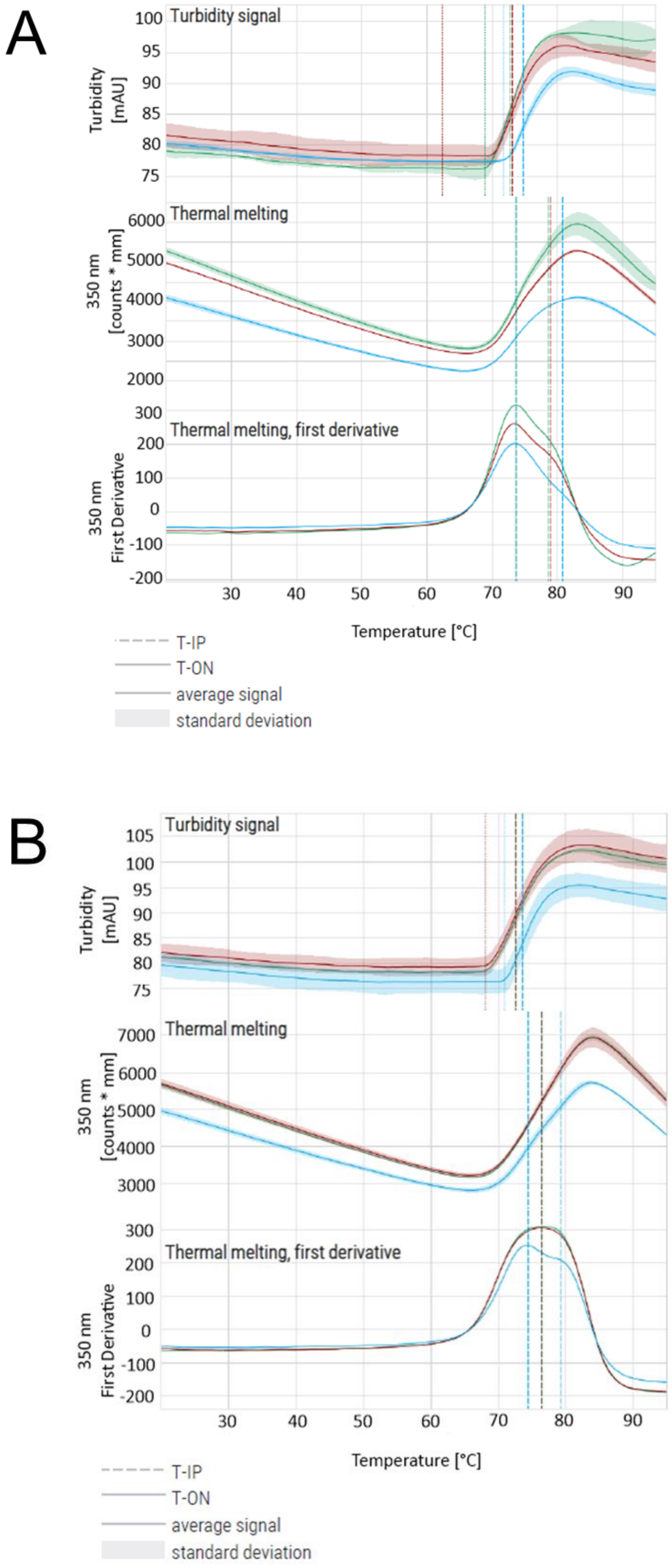

Suppl. Figure 2: **NanoDSF analyses of RTX-SCR1920 (A) and OBI-SCR1920 (B), formulated differently:** (3, blue) unformulated (elution buffer Tris-Gly-HCl); (2, red) elution buffer with 1:1,200 Trehalose, 10 mM L-His, 0.05% (w/v) Kolliphor P188; (1, green) elution buffer with 1:1,200 Trehalose, 20 mM L-His, 0.05% (w/v) Kolliphor P188. The average signal (line) and the standard deviation (shading) are shown. The macroaggregation (turbidity) and thermal melting were analyzed at increasing temperatures by light scattering and absorption measurements, respectively. T-IP, solid vertical line, representing the temperature of the inflection points determined from the first derivative of the thermal melting curve; T-ON, dashed vertical line, temperature at which the onset of macroaggregation was determined by light back-scatter;

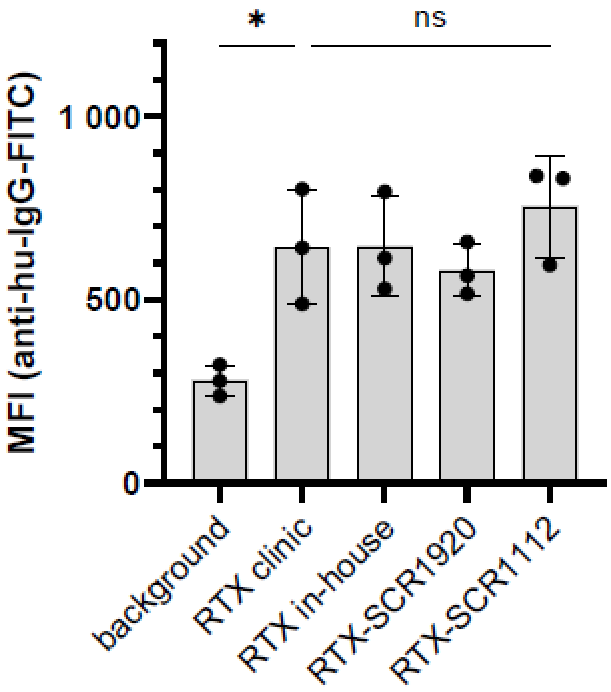

Suppl. Figure 3: The CD20 binding capacity of clinically used RTX, RTX in-house, RTX-SCR1920 and RTX-SCR1112 does not differ significantly. $2x10^5$ PBMCs of 3 different CLL patients were stained with 5 µg of the respective RTX-variant. Surface-bound RTX was detected with an anti-human-IgG-FITC-conjugated secondary antibody. The mean fluorescence intensity (MFI) of the positive population was determined and compared by performing ordinary one-way ANOVA and Tukey's multiple comparison test. ns, not significant; background, only secondary antibody; n=3; means +/-SD are shown;

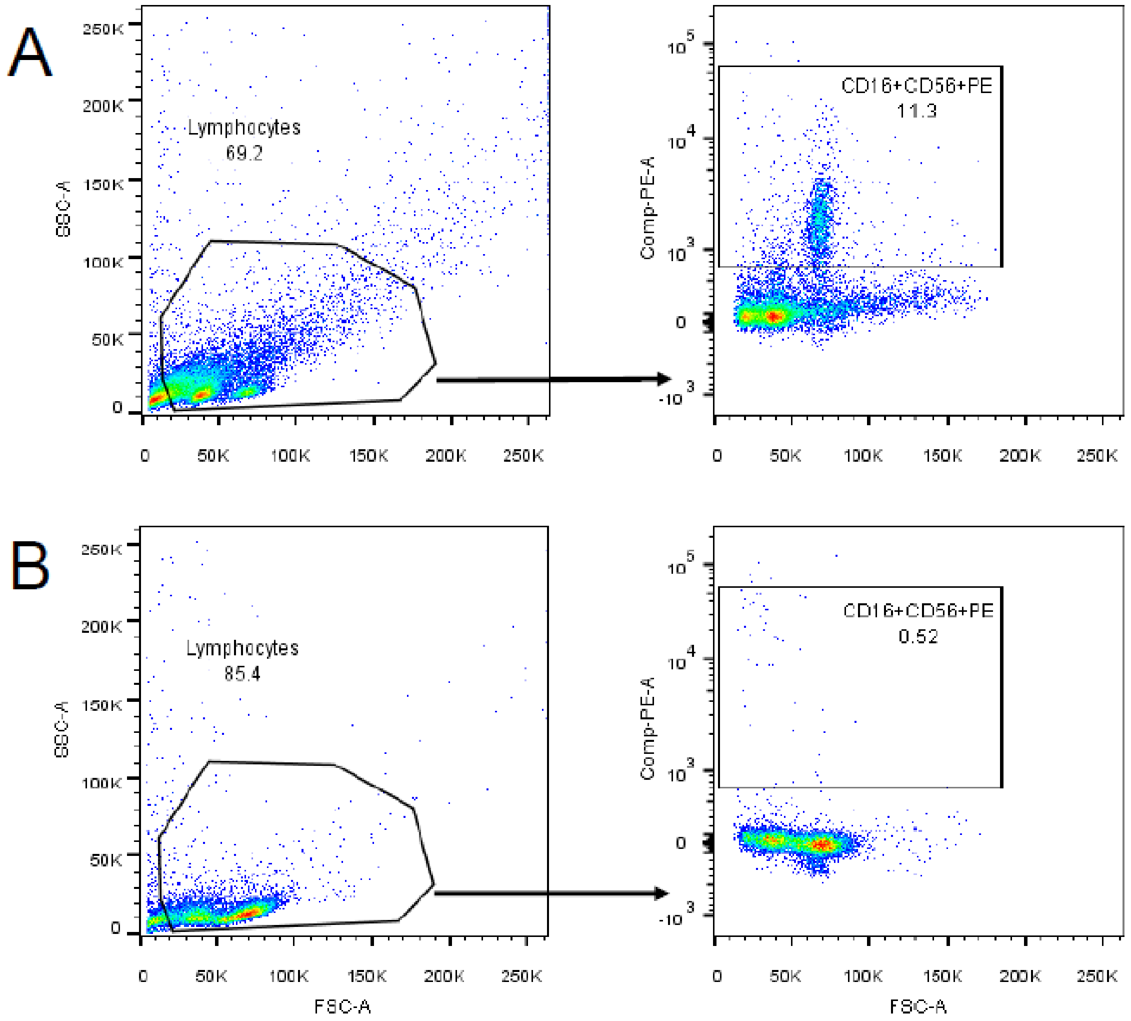

Suppl. Figure 4: The efficacy of NK cell depletion was confirmed by flow-cytometry. PBMCs of included CLL patients were analyzed pre (A) and post (B) *in vitro* NK cell depletion for the presence of CD16+CD56+ NK cells.

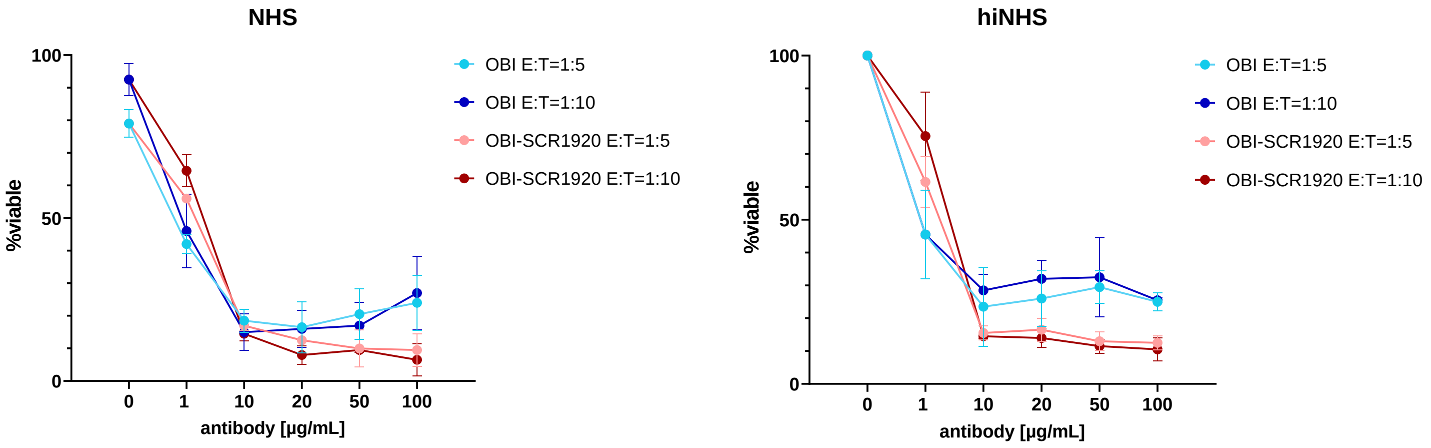

Suppl. Figure 5: OBI-SCR1920 induces increased levels of cell killing when different effector:target (E:T) ratios were tested. NK cells isolated from healthy donor blood were combined with $2x10^5$ MEC-1 cells at E:T ratios of 1:5 and 1:10. Standard CLL lysis assays with OBI or OBI-SCR1920 at concentrations ranging from 1-100 µg/mL and 20% NHS or hiNHS were performed. Viable cells were determined by PI staining and flow-cytometry. Values were normalized to a hiNHS containing control sample. n=2; means +/-SD are shown;

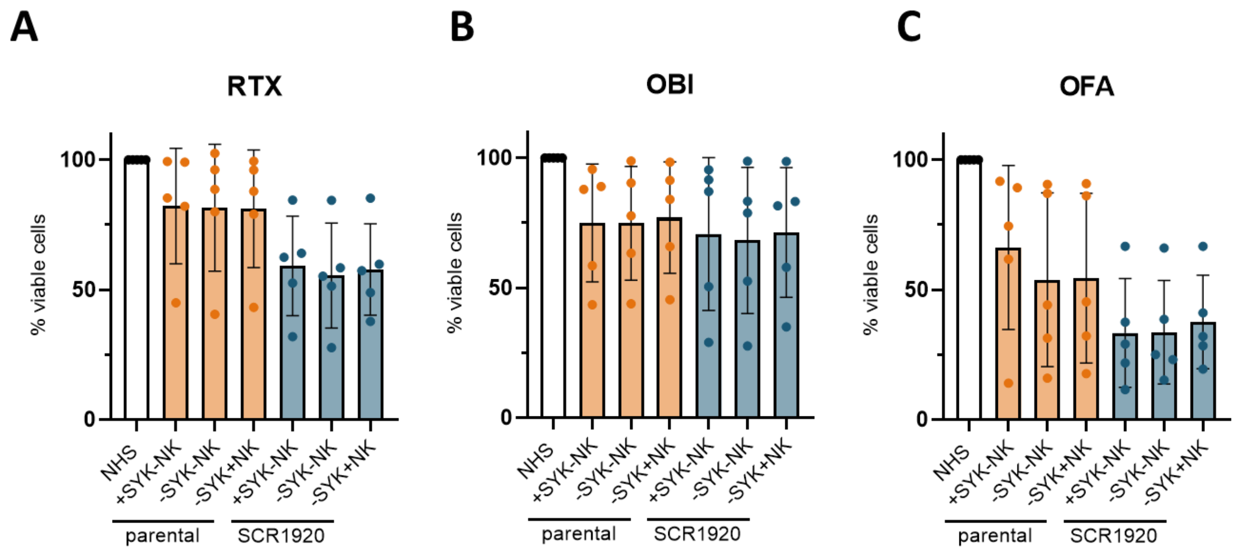

Suppl. Figure 6: Blocking phagocytosis by inhibiting the spleen tyrosine kinase (SYK) does not have an effect on the overall lysis capacity of antibody-SCR1920 conjugates. PBMCs from CLL patients were depleted of NK cells and $2x10^5$ cells per sample were analyzed in a standard CLL lysis assay with 20% NHS and 50 µg/mL of parental or SCR1920-conjugated RTX (A), OBI (B) or OFA (C) in combination with or without 1 µM SYK. Viable cells were determined by PI staining and flow-cytometry. Values were normalized to a NHS containing control sample. n=5; means +/- SD are shown;
